# Management of Patients With Glucocorticoid-Related Diseases and COVID-19

**DOI:** 10.3389/fendo.2021.705214

**Published:** 2021-09-14

**Authors:** Irina Chifu, Mario Detomas, Ulrich Dischinger, Otilia Kimpel, Felix Megerle, Stefanie Hahner, Martin Fassnacht, Barbara Altieri

**Affiliations:** ^1^Division of Endocrinology and Diabetes, Department of Internal Medicine I, University Hospital, University of Würzburg, Würzburg, Germany; ^2^Comprehensive Cancer Center Mainfranken, University of Würzburg, Würzburg, Germany; ^3^Central Laboratory, University Hospital Würzburg, Würzburg, Germany

**Keywords:** adrenal insufficiency, cushing’s syndrome, adrenocortical carcinoma, hyperaldosteronism, glucocorticoids, COVID-19, SARS-CoV-2, ACE2

## Abstract

The ongoing coronavirus disease 2019 (COVID-19) pandemic caused by severe acute respiratory syndrome coronavirus 2 (SARS-CoV-2) infection is a global health crisis affecting millions of people worldwide. SARS-CoV-2 enters the host cells by binding to angiotensin-converting enzyme 2 (ACE2) after being cleaved by the transmembrane protease serine 2 (TMPRSS2). In addition to the lung, gastrointestinal tract and kidney, ACE2 is also extensively expressed in endocrine tissues, including the pituitary and adrenal glands. Although glucocorticoids could play a central role as immunosuppressants during the cytokine storm, they can have both stimulating and inhibitory effects on immune response, depending on the timing of their administration and their circulating levels. Patients with adrenal insufficiency (AI) or Cushing’s syndrome (CS) are therefore vulnerable groups in relation to COVID-19. Additionally, patients with adrenocortical carcinoma (ACC) could also be more vulnerable to COVID-19 due to the immunosuppressive state caused by the cancer itself, by secreted glucocorticoids, and by anticancer treatments. This review comprehensively summarizes the current literature on susceptibility to and outcome of COVID-19 in AI, CS and ACC patients and emphasizes potential pathophysiological mechanisms of susceptibility to COVID-19 as well as the management of these patients in case of SARS-CoV-2. Finally, by performing an *in silico* analysis, we describe the mRNA expression of *ACE2, TMPRSS2* and the genes encoding their co-receptors *CTSB*, *CTSL* and *FURIN* in normal adrenal and adrenocortical tumors (both adenomas and carcinomas).

## 1 Introduction

The ongoing coronavirus disease 2019 (COVID-19) pandemic has changed the world. This pandemic, caused by the severe acute respiratory syndrome coronavirus 2 (SARS-CoV-2) infection, is probably the most relevant global health crisis in almost a century (1, 2). Its wide spectrum of clinical manifestations ranges from silent or mild clinical symptoms (including fever, cough, myalgia, and fatigue), which are observed in most cases, to severe forms characterized by atypical pneumonia and acute respiratory distress syndrome (ARDS), with an increased mortality risk (3). At the latest update of July 2021, over 190 million cases of COVID-19 infections and more than 3 million COVID-19-related deaths had been recorded globally (https://coronavirus.jhu.edu).

The critical aspect of SARS-CoV-2 is its efficient person-to-person transmission, rather than the mortality rate *per se* (2). SARS-CoV-2 enters the host cells by binding to angiotensin-converting enzyme 2 (ACE2) on the surface of the target cells. This interaction is promoted by the cleavage of the transmembrane spike glycoprotein, mostly by the transmembrane protease serine 2 (TMPRSS2), and by other co-receptors such as cathepsin B and L and furin (4, 5). Thus, the expression of ACE2 and TMPRSS2 on the target cells is essential for the stable binding of SARS-CoV 2.

The lungs are the main target of SARS-CoV-2, due to the abundant presence of both ACE2 and TMPRSS2. However, ACE2 and TMPRSS2 are widely distributed throughout the human body, including endocrine tissues such as the pituitary, thyroid and adrenal glands, gonads, pancreatic islets, and adipocytes (6, 7). Endocrine tissues could therefore be a direct target of SARS-CoV-2 (8). The inflammatory cascade seen with this viral infection might also induce immune-mediated glandular damage secondary to antibody formation or cell-mediated damage (9). Moreover, hormonal and metabolic conditions may influence the outcome of COVID-19 (10).

One of the most encouraging results regarding supportive therapy in patients with COVID-19 came from the RECOVERY trial. This pointed towards the effectiveness of dexamethasone in hospitalized patients receiving oxygen or invasive mechanical ventilation, whereas no benefit was observed in patients with no critical symptoms (11). This study underlines the importance of symptom-specific treatment for COVID-19, where glucocorticoids could play a central role in immunosuppression during the acute cytokine storm. However, depending on the timing of glucocorticoids administration, this immunosuppressive effects, might be beneficial (late, in cases of hyperactivation) or detrimental (when inhibiting the immune response to early) (12). Their use should thus take into account the two different pathophysiological phases of the infection: 1) the early phase, where supraphysiological doses of glucocorticoids could actually increase the plasma viral load, due to immunosuppression, and 2) the second phase, where patients might benefit from glucocorticoids to overcome hyperinflammation and the cytokine storm (13). The contrasting stimulating and inhibitory effects of glucocorticoids on immune response could increase susceptibility to SARS-CoV-2 and favor the development of severe complications in patients with impaired or excessive endogenous cortisol secretion. For this reason, patients with adrenal insufficiency (AI) or Cushing’s syndrome (CS) are considered vulnerable to COVID-19. Additionally, patients with adrenocortical carcinoma (ACC) might be particularly at risk, due to the immunosuppressive state caused by the cancer itself, glucocorticoid excess, and anticancer treatments.

This review summarizes the current literature on susceptibility to and the outcome of SARS-CoV-2 infection in patients with endocrinological diseases associated with cortisol excess or inadequate cortisol secretion, including AI, CS and ACC. The potential pathophysiological mechanisms of the increased susceptibility to COVID-19 are also discussed, as well as the management of these patients following SARS-CoV-2 infection. We also performed an *in silico* analysis to evaluate the normalized mRNA expression of *ACE2*, *TMPRSS2*, *CTSB* (encoding cathepsin B), *CTSL* (encoding cathepsin L), and *FURIN* (encoding furin), both in normal adrenal glands (n=10) and in adrenocortical tumors (22 adenomas and 33 ACC) based on the high density oligonucleotide array data published by Giordano et al. (14) (deposited in the National Center for Biotechnology Information’s Gene Expression Omnibus, accession number GSE10927).

## 2 The Hypothalamic-Pituitary-Adrenal Axis (HPA) and the Expression of ACE2 and TMPRRSS2

**Figure 1:**
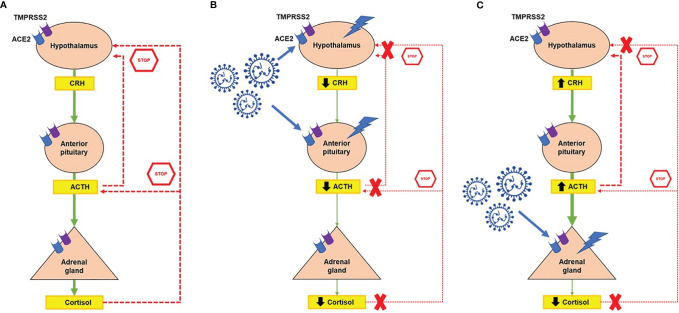
Potential interaction between the hypothalamic-pituitary-adrenal axis (HPA) and SARS-CoV-2. **(A)** Normal function of the HPA axis in response to physiological cues and stress. The paraventricular nucleus of the hypothalamus releases corticotropin-releasing hormone (CRH), which stimulates the secretion of adrenocorticotropin hormone (ACTH) from the pituitary gland. Subsequently, ACTH stimulates the synthesis and release of cortisol from the adrenal cortex. In a negative feedback loop, cortisol inhibits further release of both ACTH and CRH, whereas ACTH inhibits CRH secretion. Angiotensin-converting enzyme 2 (ACE2) and transmembrane protease serine 2 (TMPRSS2) receptors are expressed at all levels of the HPA axis. **(B)** The hypothalamus and pituitary could be directly damaged by SARS-CoV-2, resulting in central hypocortisolism. **(C)** Direct damage of the normal adrenal gland secondary to SARS-CoV-2, which might result in primary adrenal insufficiency. In all cases of adrenal insufficiency, the negative feedback loop is compromised. Symbols: ↑, increased; ↓, decreased.

The release of cortisol from the adrenal glands is regulated in a circadian manner by the hypothalamic-pituitary-adrenal (HPA) axis, in response to physiological cues and stress (**Figure 1A**) (15). The para-ventricular nucleus of the hypothalamus releases corticotropin-releasing hormone (CRH). This acts on the anterior pituitary, stimulating the corticotrope cells to secrete adrenocorticotropic hormone (ACTH). Subsequently, ACTH acts on the adrenal cortex, stimulating the synthesis and release of cortisol. In a negative feedback loop, cortisol inhibits further release of both ACTH and CRH. ACTH also provides negative feedback, inhibiting CRH secretion (**Figure 1A**).

The HPA axis plays key roles in basal homeostasis and in the body’s response to stress (16, 17). However, long-term stimulation of the HPA axis and overexposure to cortisol lead to immunosuppression and excessive catabolism. Dysregulation of the HPA axis has been associated with various disorders, including CS or AI.

**Figure 2:**
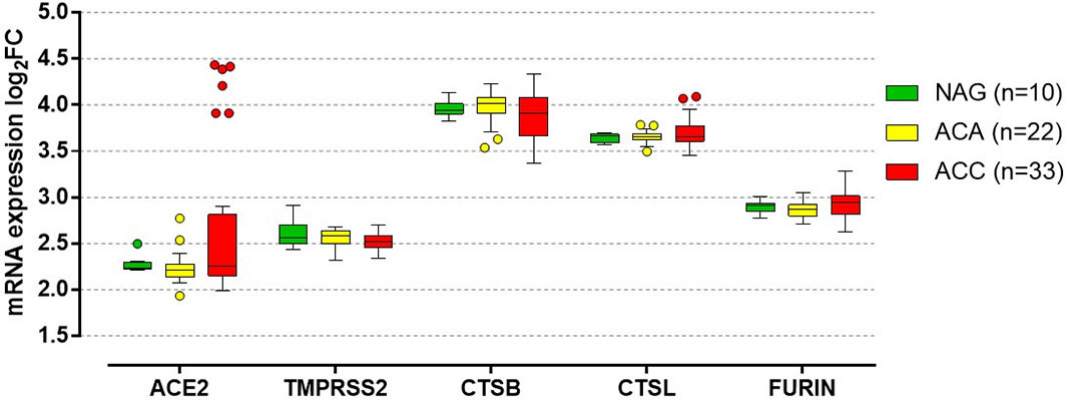
*In silico* analysis of genes involved in the SARS-CoV-2 cell entry mechanism in normal adrenal gland and adrenocortical tumors. *In silico* analysis from the high density oligonucleotide array data published by Giordano et al. (14) (deposited in the National Center for Biotechnology Information’s Gene Expression Omnibus, accession number GSE10927), evaluating the quantile-normalized and then log transformed mRNA expression of *ACE2* (encoding the angiotensin-converting enzyme 2), *TMPRSS2* (encoding the transmembrane protease serine 2), *CTSB* (encoding cathepsin B), *CTSL* (encoding cathepsin L), and *FURIN* (encoding furin) in normal adrenal glands (NAG, n=10), adrenocortical adenomas (ACA, n=22) and adrenocortical carcinomas (ACC, n=33). No significant differences between NAG, ACA and ACC were observed in the expression of the evaluated genes (*per* trend: *P=0.29* for *ACE2*, *P=0.14* for *TMPRSS2*, *P=0.39* for *CTSB*, *P=0.87* for *CTSL*, *P=0.17* for *FURIN*). In details, median log_2_ fold chance (FC) of *ACE* was: 2.24 (2.22-2.30) for NAG, 2.21 (2.14-2.27) for ACA and 2.26 (2.15-2.82) for ACC. Median log_2_FC of *TMPRSS2* was: 2.56 (2.49-2.70) for NAG, 2.59 (2.50-2.63) for ACA and 2.52 (2.45-2.58) for ACC. Median log_2_FC of *CTSB* was: 3.94 (3.90-4.01) for NAG, 4.01 (3.91-4.08) for ACA and 3.91 (3.66-4.08) for ACC. Median log_2_FC of *CTSL* was: 3.66 (3.59-3.68) for NAG, 3.65 (3.62-3.68) for ACA and 3.65 (3.60-3.77) for ACC. Median log_2_FC of *FURIN* was: 2.91 (2.85-2.93) for NAG, 2.86 (2.80-2.92) for ACA and 2.94 (2.81-3.01) for ACC. Data are reported as median with the 25% and 75% percentile. For the details on the quantile-normalization procedures and the log transformed of the array data, see the published paper by Giordano et al. (14). Statistical analysis performed by Kruskal-Wallis test using GraphPad Prism (version 5.0, La Jolla, CA, USA).

ACE2 and TMPRSS2 have been identified at all levels of the HPA axis (**Figure 1**) (6). Autopsy findings confirmed that the hypothalamus is a strong potential target of SARS-CoV-2, due to the high expression of both ACE2 and TMPRRS2, particularly in the para-ventricular nucleus (18). Moreover, brain edema and neurological degeneration have been found in patients with COVID-19 (19, 20), confirming that SARS-CoV-2 could enter the brain cells, including in the hypothalamus and pituitary. More recently, the expression of ACE2 receptors has been demonstrated in the zona fasciculata and in the zona reticularis of the adrenal cortex by immunohistochemistry (21). By performing an *in silico* analysis of Giordano et al.’s high density oligonucleotide array data (14), we were able to show that normal adrenal glands express both *ACE2* and *TMPRSS2* and the genes coding for their co-receptors cathepsin B (*CTSB*), cathepsin L (*CTSL*), and furin (*FURIN*) (**Figure 2**), which are involved in the cleavage of the SARS-CoV-2 spike glycoprotein before it enters the host cells. Moreover, similar slightly elevated mRNA levels of these genes were present in benign and malignant adrenocortical tumors (**Figure 2**). All these findings suggest that the entire HPA axis could be a direct target of SARS-CoV-2 (**Figures 1B, C**).

## 3 Adrenal Insufficiency

AI is a rare endocrine disorder characterized by impaired cortisol synthesis. It can be caused by primary adrenal failure (primary adrenal insufficiency, PAI), by impaired release of ACTH from the pituitary gland (secondary adrenal insufficiency, SAI) or by reduced CRH secretion by the hypothalamus (tertiary adrenal insufficiency, TAI) (22, 23). Autoimmune adrenalitis is the most common form of PAI in developed countries, followed by infectious diseases (tuberculosis, HIV), genetic causes (congenital adrenal hyperplasia, adrenoleukodystrophy), destruction of the adrenal gland (hemorrhage, neoplasia, infiltrative disease) and iatrogenic causes (adrenalectomy, steroidogenesis inhibitors). SAI is caused by pituitary tumors and their treatments (surgery, radiation), autoimmune hypophysitis, trauma, infiltrative diseases, pituitary apoplexy or rare genetic causes. TAI is usually the result of long-term use of glucocorticoids (22–24).

Without adequate treatment, AI is a life-threatening condition. Replacement with glucocorticoids should be initiated immediately upon diagnostic confirmation, or even before the diagnosis can be confirmed, where there is a strong clinical suspicion (25). PAI patients also require life-long mineralocorticoid replacement with mineralocorticoids (25). Hydrocortisone is the drug of choice for glucocorticoid replacement therapy (26). Due to its short plasma half-life (approx. 90 minutes) it is usually given twice or thrice daily, guided by the circadian rhythm of cortisol secretion (two thirds of the total daily dose in the morning, one third in the early afternoon) (22, 24, 25). However, pronounced fluctuations of serum cortisol ranging from supraphysiological to almost undetectable levels have been reported with this therapy regimen (27, 28). A modified-release hydrocortisone is designed to more closely resemble the physiological cortisol profile (29). This modified hydrocortisone seems to have beneficial effects on the cardiometabolic and immune profile of AI patients compared with hydrocortisone (26, 29, 30). Furthermore, its improved bioavailability over time leads to a ~20% reduction in the total daily cortisol exposure (29). It is still a matter of debate whether the metabolic and immunologic improvements result from the different release pattern or from the lower cortisol dose. Another option is the intermediate-acting glucocorticoid prednisolone, which can be administered once daily due to its longer half-life (25, 28). However, prednisolone has been linked to adverse cardiometabolic effects (dyslipidemia, increased bone turnover) and should thus be used cautiously in AI patients (31, 32).

Patient education plays a major role in the long-term management of AI, especially in the prevention and treatment of life-threatening adrenal crises. Despite continuous efforts to improve patient awareness, adrenal crisis is still a common occurrence (4-15/100 patient-years) and is fatal in up to 6.3% of cases (33–35). Infections, especially gastroenteritis, bronchopulmonary infections and urinary tract infections, are still the most common triggering event for adrenal crises (33–36). Interestingly, the highest incidence of adrenal crises was reported for a small cohort of 28 TAI patients (15/100 patient-years) (33).

In relation to AI in the context of the COVID-19 pandemic, two issues need to be addressed:

1. the risk of severe forms of COVID-19 infection and the management of patients with known AI and symptomatic SARS-CoV-2 infection; and
2. the risk of *de novo* development of AI during the course of or even due to COVID-19.

### 3.1 Risk and Management of Patients With Known AI Under Established Replacement Therapy With Glucocorticoids

Previous studies have reported an increased risk of “common” infections as well as increased infection-related hospitalization and mortality rates in AI patients compared to controls (37, 38). Therefore, we would expect to see a similar pattern with COVID-19 (39). One possible explanation for the increased infection risk in AI patients lies in their impaired immune response to viral infections and altered immune profile under classic hydrocortisone replacement therapy (26, 40). Hydrocortisone fails to reproduce the circadian rhythm of cortisol secretion, which is known to have important metabolic and immunoregulatory effects (41–43). The peaks and troughs associated with hydrocortisone administrations might therefore impair immunity, as suggested by the extreme inflammatory response in adrenal crises as well as the increased susceptibility to infections (40). Switching “classic” hydrocortisone to a modified release hydrocortisone not only provides a more physiological circadian cortisol profile but also restores the immune alterations and lowers the infection rate seen with standard hydrocortisone (26). However, the increased infection risk seen in AI patients might also be partially explained by bias, since these patients might seek medical attention more often compared to the general population. In the event of signs of infection AI patients are in fact encouraged to contact health care providers without delay if they are unsure about the need for glucocorticoid dose adjustments, and are also more likely to experience impaired general health requiring hospital admission.

Information on the prevalence of COVID-19 in AI patients is, in any case, scarce, making it difficult to draw any conclusions on the risk and severity of the disease in this patient population. This could be explained in part by the relatively low number of symptomatic patients who have actually been tested (44–46). According to a retrospective case-control study enrolling 279 patients with AI and 112 controls, nasopharyngeal swabs were performed in only 12 out of 92 symptomatic participants (8 AI, 4 controls) (44). Similarly, in a cross-sectional study including 159 patients with secondary AI, only 7 out of 30 symptomatic participants were actively tested (46). COVID-19 was confirmed in 2 AI patients in each study. Therefore, the prevalence of COVID-19 in this patient population might be underestimated as a result of the screening approach. Additionally, strict implementation of safety protocols might have efficiently reduced exposure to SARS-CoV-2 among AI patients.

Nevertheless, there was no difference between AI patients and controls in the severity of viral infections recorded during the COVID-19 pandemic (44). Moreover, no adrenal crises and no need for hospitalization were reported by symptomatic AI patients, regardless of whether or not they were screened for COVID-19 (44–46). Compared to controls, AI patients had a shorter duration of symptoms of viral infection and a lower prevalence of gastrointestinal symptoms (44). Two different studies reported increases in the daily glucocorticoid dose in about one third of the enrolled AI patients, not only due to symptoms of infection or confirmed COVID-19 but also due to pandemic-related stress (44, 45). These observations might simply reflect patient awareness and proper patient education, resulting in adequate adjustment of glucocorticoids when necessary. Dose adjustments might help lessen the severity of COVID-19 by suppressing the inflammatory response seen with severe cases.

The negative long-term impact that glucocorticoid replacement might have on immunity in AI should certainly not prevent patients (or their physicians) from increasing their glucocorticoid dose in the event of an acute infection, the most common trigger of adrenal crises (33, 34, 36, 37, 47, 48). In healthy subjects, infection induces a significant increase in cortisol bioavailability, thus preventing the deleterious effects of an extreme inflammatory reaction (49). This physiological increase in cortisol is absent in AI patients and must therefore be imitated by increasing exogenous glucocorticoids as soon as possible.

SARS-CoV-2 is a highly pathogenic virus that causes severe forms of COVID-19 in up to 20% of cases, due to hyperinflammation (50–53). An extreme inflammatory response has been associated with higher rates of intensive care unit (ICU) admission, invasive ventilation and mortality (53). Therefore, we could expect worst-case scenarios in AI patients, due to the additional lack of the immunosuppressive effects of glucocorticoids.

Experts from different countries have reacted promptly to this issue by publishing recommendations on the management of steroid replacement in AI patients during the COVID-19 pandemic (13, 39, 54, 55). The European guidelines for the management of AI under COVID-19 pandemic conditions recommend: 1) administering 20 mg hydrocortisone orally every 6 hours in case of “signs and symptoms suggestive of COVID-19” (sick day rule 1), and 2) in the event of clinical deterioration, that patients immediately self-inject 100 mg hydrocortisone intramuscularly and contact the emergency services for transfer to hospital (sick day rule 2) (39, 56). Modified release hydrocortisone should be switched to short-acting hydrocortisone, whereas long-acting glucocorticoids like prednisolone should be split into a morning and late afternoon dose consisting of at least 10 mg each (39). Even if the replacement therapy with 20 mg hydrocortisone every 6 hours results in variable cortisol levels with alternating periods of relatively low followed by high cortisol levels, it still provides patients with a better glucocorticoid coverage over 24 hours and improves the early morning nadir seen in classic glucocorticoid replacement strategies, where the last dose is taken in the early afternoon (56).

Ensuring that AI patients receive the necessary education regarding “sick day rules” and are equipped with an emergency pass, an emergency kit, and adequate supplies of immediate-release glucocorticoids is mandatory for their optimal self-management in the event of an imminent adrenal crisis (25, 39, 55). Replacing live training sessions with telephone or online meetings to enable compliance with social distancing rules and recommendations is a feasible solution to enable all AI patients to receive the essential information (55). However, this does not cover one of the most important aspects of the training, namely practicing the self-administration of the hydrocortisone emergency injection under the direct supervision of qualified medical staff. For this reason “classic” training sessions, adapted as necessary (reducing the number of participants and the session time, mandatory wearing of face masks, keeping an appropriate distance between participants, ventilation, etc.), should be preferred where possible (55).

Special consideration should be given to older patients (>60 years) with AI, as they are more susceptible to infections and hospital admissions (47). Comorbidities, cognitive impairment and isolation may significantly impair the self-management of elderly patients and their recourse to medical care if their health deteriorates (57). Moreover, signs and symptoms of adrenal crises might be misclassified as exacerbations of other comorbidities or adverse effects of their medication. For this reason, not only elderly AI patients but also their family members, legal guardians and/or general practitioners should be involved in the educational process and trained to recognize and manage adrenal crises (57).

### 3.2 *De Novo* Development of AI During a SARS-CoV-2

The HPA axis may be affected at different levels and by different mechanisms by SARS-CoV-2 (**Figure 1**).

#### 3.2.1 Central Hypocortisolism

Patients with SARS-CoV-2 might be at risk of developing central hypocortisolism, as seen with SARS-CoV-1 (SARS), as the two viruses share ~80% genetic homology (8, 58, 59). SARS has been associated with transient central hypocortisolism, as a possible result of either hypophysitis or hypothalamic damage (58). Moreover, the molecular mimicry between SARS and ACTH might stimulate the production of ACTH-antibodies as part of an immune-evasive strategy (8, 59, 60). Considering the presence of ACE2 and TMPRSS2 receptors in the hypothalamus and pituitary (6, 18), we can expect the changes in the functionality of the HPA axis induced by SARS-CoV-2 to be similar to those seen with SARS-CoV-1 (**Figure 1B** and **Table 1**).

**Table 1:** Potential mechanisms involved in *de novo* development of adrenal insufficiency due to SARS-CoV-2.

HPA levels	Potential mechanisms
Hypothalamus and pituitary gland damage	- Immune-mediated damage to the hypothalamus and/or pituitary gland, resulting in impaired release of CRH and/or ACTH. - Production of ACTH autoantibodies resulting in impaired ACTH release due to molecular mimicry between SARS-CoV-1 (and therefore probably also SARS-CoV-2) and ACTH.
Adrenal gland damage	- Ischemic necrosis/infarction (often bilateral). - Hemorrhage. - Cortical lipid degeneration. - Focal inflammation.

ACTH, adrenocorticotropic hormone; CRH, Corticotropin-releasing hormone; HPA, hypothalamic-pituitary-adrenal axis; SARS, Severe Acute Respiratory Syndrome.

So far, evidence of central hypocortisolism in patients with COVID-19 points towards critical illness-related corticosteroid insufficiency (CIRCI), also known as relative adrenal insufficiency. CIRCI describes glucocorticoid levels that are inadequately low for the severity of the critical illness, resulting in a magnified systemic inflammatory response (61, 62). It occurs most commonly in septic shock and ARDS and is associated with a prolonged need for intensive care as well as increased morbidity and mortality (61, 62). Mechanisms leading to CIRCI include disrupted CRH/ACTH and cortisol synthesis, reduced cortisol metabolism, and glucocorticoid resistance (61, 62). Biochemically, CIRCI is defined as a random plasma cortisol of <10 μg/dl (275 nmol/l) or an increase in cortisol of <9 µg/dl (248 nmol/l) 60 minutes after stimulation with 250 µg cosyntropin (61, 62). Administration of glucocorticoids is only recommended in patients with septic shock that is refractory to fluid and vasopressor therapy (61, 62). Several studies reported low cortisol levels, meeting CIRCI criteria (<10 µg/dl), in hospitalized patients with COVID-19. Both low morning cortisol (median 196 nmol/L) and low corresponding ACTH levels (median 18.5 ng/L) were seen during the acute phase of COVID-19 in 28 consecutive patients who had not received prior glucocorticoid treatment and had no medical conditions that might affect adrenal function (63). These results were confirmed in up to 60% of cases on repeat testing, suggesting a form of secondary AI (63). Moreover, a higher prevalence of CIRCI was seen in critically ill patients diagnosed with COVID-19 compared to critically ill non-COVID-19 patients, as well as in COVID-19 patients with a more severe form of the disease (21, 63). Critically ill patients with COVID-19 also often require vasopressor support (22-67%), as seen with CIRCI (21, 61).

However, these results are in contrast with a previous study reporting a higher median random cortisol concentration during the first 48 hours after hospital admission in patients with confirmed or highly suspected COVID-19 infection (n=403, 619 nmol/L) compared to non-COVID-19 patients (n=132, 519 nmol/L) (64). Furthermore, in this study there was a significant correlation between mortality and increasing cortisol levels, even after adjusting for potential confounding factors. These contradictory results might be explained by different patient populations, as well as different strategies for assessing adrenal function. Increased cortisol levels do not necessarily describe the activation of the HPA axis, as they can also result from reduced cortisol metabolism or low levels of cortisol binding globulin. Furthermore, increased cortisol levels in the acute phase of COVID-19 might be an indicator of disease severity and do not exclude the possible onset of relative AI at some later point. Moreover, pooling the results from patients with confirmed COVID-19 infection and from patients suspected of COVID-19 despite negative real-time RT-PCR testing might falsely increase the median cortisol level reported for the whole group.

Suppression of the HPA axis in COVID-19 patients could also result from treatment with corticosteroids. Short-term treatment with high doses of dexamethasone has been successfully used in COVID-19 patients requiring respiratory support (11). Dexamethasone, however, belongs to the class of most potent, long-acting glucocorticoids. Its strong anti-inflammatory effect reflects its ability to suppress the HPA axis. The risk of tertiary AI generally increases with the dose, duration, systemic application and potency of the chosen glucocorticoid, but cases of AI have also been registered with low-dose and/or short-term therapies (65, 66). To our knowledge, no data on the incidence of hypocortisolism in COVID-19 patients following glucocorticoid therapy have been published.

Overall, data on the incidence and clinical relevance of hypocortisolism following corticosteroid therapy are limited to small and highly heterogeneous observational studies. This heterogeneity makes it difficult to reach a consensus on whether and how to perform glucocorticoid tapering and when and how to test adrenal function. The ongoing TOASST study (NCT03153527) will hopefully provide better insights into the feasibility and safety of abrupt termination of glucocorticoid therapy. This prospective, placebo-controlled study assesses the incidence of adrenal crises following abrupt cessation of treatment with glucocorticoids compared to a standardized tapering regime over 4 weeks in patients treated for at least 28 days with a cumulative glucocorticoid dose of ≥420 mg prednisone-equivalent who were receiving at least 7.5 mg prednisone-equivalent at the time of inclusion.

Special consideration should also be given to patients who are generally at risk of AI due to opioids, drugs affecting glucocorticoid bioavailability, or immune-modulating therapies (67). AI might become manifest more often than expected in this population, which is already susceptible to hypocortisolism, where COVID-19 might act like a “second blow”.

#### 3.2.2 Primary Adrenal Insufficiency

Our *in silico* analysis (14) confirmed the expression of *ACE2* and *TMPRSS2* mRNA as well as of *CTSB*, *CTSL*, and *FURIN* mRNA in normal adrenal tissues (**Figure 2**). Moreover, positive immunostaining of ACE2 receptors has been detected in the zona fasciculata and zona reticularis of the adrenal cortex (21). The adrenal glands are thus a potential direct target of SARS-CoV-2 (**Figure 1C**) and primary AI might occur in COVID-19 patients as a result of direct vascular and structural damage to these glands (68, 69) (**Table 1**). Autopsies performed in a series of 28 cases with severe forms of COVID-19 revealed macro- and microscopic adrenal lesions in 12 patients (43%) (69). Necrosis was the most prevalent lesion (7/12), which is consistent with the high incidence of acute adrenal infarction (23%) seen in a retrospective analysis of CT scans from patients with severe forms of COVID-19 (69, 70). Adrenal infarction was bilateral in the majority of cases (45/51) and was associated with a higher degree of lung injury, a higher rate of ICU admission and a longer hospital stay (70). Based on a biochemical triad suggestive of hypocortisolism (hyponatremia + hyperkalemia + hypoglycemia), AI was suspected in 4 patients with bilateral adrenal infarction (70). However, none of the patients with adrenal injuries from the autopsy study had plasma cortisol levels consistent with CIRCI, i.e. <10 µg/dl (69). Unfortunately, ACTH levels were not assessed in any of these studies. The ongoing prospective study (ChiCTR20000301150) evaluating both serum cortisol and ACTH levels in COVID-19 patients is expected to give a better insight into the prevalence and etiology of COVID-19-associated AI (59).

Even though ACE2 receptors have been detected in the adrenal cortex, no data are available regarding the presence of viral antigens or genomic sequences in the adrenal gland. Therefore, we cannot draw any conclusions at this stage on whether the detected adrenal lesions are caused by a direct effect of COVID-19 or by non-specific organ damage due to critical illness.

## 4 Cushing’s Syndrome

CS, characterized by clinically significant endogenous hypercortisolism, is a rare disorder with an incidence of 1.2-2.4 per million person-years (71, 72). Only seven patients with CS and COVID-19 are reported in the literature (73–76), with a prevalence ranging from 3.2% to 13.6% (74, 76). Apart from one case of suspected ectopic CS (76), in all other cases the origin of the hypercortisolism was an ACTH-secreting pituitary adenoma, also called Cushing’s disease (CD). The low prevalence of SARS-CoV-2 in patients with CS might be explained merely by the rarity of the underlying disease, or could suggest a lower susceptibility to SARS-CoV-2 among these patients.

Two patients with mild hypercortisolism due to CD presented with an asymptomatic or mild clinical form of COVID-19 despite also suffering from comorbidities, including type 2 diabetes mellitus (T2DM), obesity and hypertension (74, 76). Neither of these patients were receiving any medication for their hypercortisolism at the time of their COVID-19 diagnosis. Only one, a 66-year-old woman, was hospitalized to prevent possible complications (76).

In contrast, two women with CD, aged 27 and 38, presented moderately elevated cortisol levels when they were tested positive for SARS-CoV-2 (75, 76). Although neither had any other comorbidities associated with severe forms of COVID-19, both required hospitalization and treatment with oxygen and antibiotics. They were both discharged after 21-24 days and experienced full recovery (75). A ‘block and replace’ regimen with metyrapone, trilostane and hydrocortisone was used in the management of the 27-year-old patient. Patients with active CD under medical treatment might generally be at higher risk of AI crises due to the impaired increase in cortisol secretion in response to infections or other stress situations (73). The signs and symptoms of hypocortisolism should therefore be carefully assessed during medical treatment for CS (77). If hypocortisolism is suspected, glucocorticoid replacement should be initiated or adjusted, whereas the medication aimed at controlling the endogenous hypercortisolism should be maintained (“block and replace”).

In two out of seven cases of CD (28.5%), fulminant progression of COVID-19 pneumonia causing death was reported (74, 76). The first case was a 55-year-old woman with history of CD in remission taking glucocorticoid replacement therapy due to secondary AI, who died after 6 days of hospitalization (74). However, this patient also had end-stage chronic kidney disease and malnutrition, which probably contributed to the severe progression of COVID-19. The second case was a 71-year-old woman with active severe hypercortisolism, probably caused by an ectopic ACTH-secreting tumor of unknown origin (76). She died 7 days after SARS-CoV-2 was confirmed by RT-PCR, due to bilateral polysegmental hemorrhagic pneumonia and ARDS.

Given that few cases of SARS-CoV-2 have been reported in patients with hypercortisolism, it is difficult to draw definitive conclusions regarding the contribution of endogenous cortisol excess to the severity and outcome of COVID-19 in such patients. Based on the reported cases, it seems that the clinical course of COVID-19 depends on the severity of the endogenous hypercortisolism, as seen in acute stress response conditions, where higher cortisol levels are associated with COVID-19-related mortality (64). Overall, reasoning in terms of the direct and indirect effects of cortisol on the body, we can assume that endogenous hypercortisolism might increase susceptibility to COVID-19, for the following reasons.

It is well known that over time, high doses of glucocorticoids suppress the innate and adaptive immune system (78) (**Table 2**). The main effects of high cortisol levels in CS patients are leukocytosis, neutrophilia, lymphocytopenia, and monocytopenia (78). Although the overall number of neutrophils is increased, their ability to migrate to damaged tissues and their response to inflammatory stimuli such as interleukin 8 (IL-8), tumor necrosis factor α (TNFα) and interleukin 1β (IL-1β) are impaired (79, 80). Hypercortisolism also affects the activation of natural killer cells and the complement system (78, 105, 106). All these changes can interfere with the immune response to viral infections.

**Table 2:** Cushing’s syndrome comorbidities and possible interactions with COVID-19.

Comorbidities related to Cushing’s syndrome	Effects of high cortisol levels	Possible risks in COVID-19 patients
Immune system modification	- Leukocytosis and neutrophilia (78) - ↓ B-lymphocytes and T-lymphocytes (78) - ↓ Neutrophil response to IL-8, TNF-α and IL-1β (79, 80) - ↑ Proinflammatory cytokines secretion (78, 81)	- Immune system modification increases the risk of viral infections - Pro-inflammatory state of immune system modification leading to ARDS in case of COVID-19 (82)
Obesity	- ↑ IL-1, IL-6 and TNF-α (83, 84) - ↑ Number of adipocytes (85)	- Pro-inflammatory state of obesity leading to ARDS in case of COVID-19 (82) - Being adipocytes SARS-CoV-2 reservoir (thanks to ACE2 receptor) (86)
Type 2 diabetes mellitus	- Worsening hyperglycemia and ↑ DPP-4 (87)	- DPP-4 is a surface receptor for SARS-CoV-2 infection (87, 88) - Worsening of the already high hypercoagulation state associated with COVID-19 (89) - ↑ CFR in patients with diabetes and COVID-19 (90, 91)
Hypercoagulation	- ↑ Factor VIII, fibrinogen, and vWF (92, 93) - Shortening of the aPTT (92, 93) - ↓ Fibrinolysis (92, 93)	- Worsening of the already high hypercoagulation state associated with COVID-19 (94, 95)
Arterial hypertension	- Modifying the RAAS, mineralocorticoid activity, vasoregulation mechanism and sympathetic nervous system (81) - ↑ IL-17, interfering with natural killer and cytotoxic T-cell function (96)	- Arterial hypertension is a negative prognostic factor for COVID-19 (97, 98)
Bone metabolism alteration	- ↑ Osteoporosis, osteopenia and bone fractures (99, 100)	- Vertebral fractures are a negative prognostic factor for COVID-19 patients (101)
Neuropsychiatric disorders	- Major depression, anxiety and borderline disorder (102)	- Worsening of mental health during COVID-19 pandemic (103, 104)

ACE2, angiotensin converting enzyme 2; ARDS, acute respiratory distress syndrome; CFR, case fatality rate; DPP-4, dipeptidyl peptidase-4; IL, interleukin; aPTT, partial thromboplastin time; RAAS, renin-angiotensin-aldosterone system; TNF-α, tumor necrosis factor -α; vWF, von Willebrand factor; ↑, increased; ↓, decreased.

There are contrasting opinions regarding the risk of ARDS due to SARS-CoV-2 in CS patients. Prolonged hypercortisolism, in fact, can increase the secretion of proinflammatory cytokines, including IL-6 and TNF-α (78, 81), which could promote the onset of ARDS in patients with SARS-CoV-2 (82, 86). On the other hand, several observations suggest that CS is characterized by a reduced cytokine response to external agents (107), and that glucocorticoids could thus have positive effects in severe cases of COVID-19 (11, 108). Apart from the patient with ectopic CS (76), all cases presented non-severe forms of COVID-19, from which they recovered completely.

Chronic cortisol excess is associated with an increase in visceral fat and prevalence of obesity (85). Obesity represents a chronic state of hypoxia for the adipose tissue, leading to increased levels of IL-1, IL-6 and TNF-α (83, 84). This pro-inflammatory state might have a negative impact on the prognosis of COVID-19 (82) (**Table 2**). Furthermore, white adipocytes express ACE2 and could, therefore, be a reservoir of SARS-CoV-2 (86).

Other important comorbidities related to CS are hyperglycemia and T2DM, which have been associated with an increased risk of severe SARS-CoV-2 infection (109). This association could be related to several factors (**Table 2**), including impaired antiviral immunity (110, 111). Moreover, dipeptidyl peptidase-4 (DPP-4), a glycoprotein responsible for the degradation of incretins, is elevated in patients with T2DM. DPP-4 has been identified as a surface receptor for coronaviruses and is associated with the development of Middle East Respiratory Syndrome (MERS) (87, 88). Interestingly, hyperglycemia has been seen in 35-58% of patients with COVID-19, suggesting an impaired glucose metabolism (87, 112). Some studies also reported an increased fatality rate in patients with COVID-19 and diabetes mellitus, compared to individuals without diabetes (87, 89, 90).

Hypercoagulation and the risk of thromboembolic events is also a common feature of CS and COVID-19 (113, 114) (**Table 2**) and implies several mechanisms such as increased levels of factor VIII, fibrinogen, and von Willebrand factor (vWF); shorter activated partial thromboplastin time (92, 93); reduction in fibrinolysis, or elevated antiphospholipid antibodies (94, 95). It is also believed that COVID-19 patients with diabetes have a higher risk of thrombosis than non-diabetic patients (89). Therefore, we might also assume a further increase in the risk of thromboembolic events in patients with CS and COVID-19.

Systemic arterial hypertension is present in more than a quarter of patients with CS (81). A high prevalence of hypertension has also been reported in COVID-19 patients, where it seems to be correlated with a higher mortality (97, 98). It is associated with an impaired immune function with higher IL-17 levels as well as abnormal natural killer and cytotoxic T-cell function (96).

Osteopenia occurs in up to 78% of CS patients, and osteoporosis in up to 57% (81, 99, 100, 115, 116). Fractures are therefore common in these patients. Interestingly, a retrospective study evaluating the incidence of vertebral fractures in patients with COVID-19 admitted to the emergency department found that patients with vertebral fractures more frequently required hospitalization and mechanical ventilation compared to those without vertebral fractures (101). Furthermore, patients with severe vertebral fractures had a higher mortality rate than those with moderate or mild fractures (101). Impaired bone metabolism underlies another potential link between CS and the severity of COVID-19 (**Table 2**).

Finally, neuropsychiatric disorders like major depression, anxiety and bipolar disorders are already quite common in CS patients (102). Furthermore, mental health problems increased globally during the COVID-19 pandemic (103, 104). Therefore, COVID-19 could worsen the mental health of an at-risk group with neuropsychiatric disorders, like CS patients (**Table 2**).

Since all the above-mentioned comorbidities (summarized in **Table 2**) could worsen the severity of COVID-19, patients with CS and COVID-19 should be treated with standard of care, as suggested by the recent clinical practice recommendations (77). However, the beneficial effects of short-term use of glucocorticoids on the suppression of the cytokine storm and prevention of ARDS should not be disregarded out of fear of possible metabolic complications. In fact, the 28-day mortality of patients receiving invasive mechanical ventilation or oxygen alone was significantly lowered by treatment with 6 mg dexamethasone daily for up to 10 days (11). This result advocates for the short-term use of glucocorticoids in patients requiring respiratory support. The benefit of glucocorticoids could be explained by the rapid induction of glucocorticoid-induced leucine zipper (*GILZ*) (117, 118). *GILZ* mediates many glucocorticoid anti-inflammatory effects, including the inhibition of the NF-κB and MAPK pathways.

Considering the large variation in the clinical presentation of CS, it seems that the severity of the hypercortisolism and the duration of the disease are of particular importance for the individual evaluation of the risk/benefit ratio. Clinical practice recommendations suggest the use of steroidogenesis inhibitors for the treatment of most CS patients during the COVID-19 pandemic. Surgery is recommended only in cases insufficiently controlled by medical therapy, in cases of optic chiasm compression or in cases of suspected/confirmed malignancy (77). Moreover, in cases of SARS-CoV-2 infection in CS patients, the ‘block and replace’ approach is a feasible option to reduce the risk of AI crises caused by impaired HPA axis dynamics (77).

## 5 Adrenocortical Carcinoma

ACC is a rare endocrine malignancy with an estimated incidence of 0.5-2 new cases per year (119, 120). About 50-60% of ACC patients present with symptoms of hormone excess, most frequently with hypercortisolism alone or in combination with hyperandrogenism, whereas mineralocorticoid excess (Conn’s syndrome) is described in only 2-3% of cases (121).

It is generally assumed that cancer patients could be more vulnerable to SARS-CoV-2 and are at higher risk of developing severe complications, due to the immunosuppression caused by the cancer itself, its treatments, and any comorbidities (122–125). Due to the rarity of ACC, it is not surprising that only 6 ACC cases with confirmed COVID-19 infection have been reported (126). Most of these patients came from the Lombardy region in Italy, which reported the highest numbers of SARS-CoV-2 cases worldwide from February to April 2020 (127). The frequency of symptomatic SARS-CoV-2 infection was 6.5% in ACC patients compared to 2.5% in patients with other types of solid tumors followed at the same center (126). Tumor secretion of hormones was diagnosed in four of the six ACC cases, of which two were cortisol-secreting and two were androgen-secreting. All patients were treated with mitotane in an adjuvant setting (n=4) or for advanced disease (one as monotherapy and one associated with temozolomide). Mitotane was suspended in all cases until complete symptom resolution. Glucocorticoid replacement with cortisone acetate was maintained, and increased following the onset of any fever. Four patients (66.7%) developed mild or moderate symptoms, while two (33.3%) required hospitalization for acute interstitial pneumonia. Both patients requiring hospitalization had a cortisol-producing carcinoma, but none of them were hypercortisolemic at the time of the SARS-CoV-2 infection. One of them, a 60 year-old man treated with adjuvant mitotane, achieved a full recovery after treatment with hydroxychloroquine, azithromycin, antiviral therapy, non-invasive oxygen support and increased cortisol replacement with up to 300 mg hydrocortisone i.v./day during hospitalization. The second case was a 28 year-old woman with multiple lung and liver metastases, treated with mitotane plus temozolomide as a second line treatment after disease progression under the combination of a platinum compound with etoposide and doxorubicin. She presented lymphopenia before hospitalization and had serum mitotane levels above the therapeutic range (21 mg/L). She died of ARDS after 15 days of hospitalization (126).

**Figure 3:**
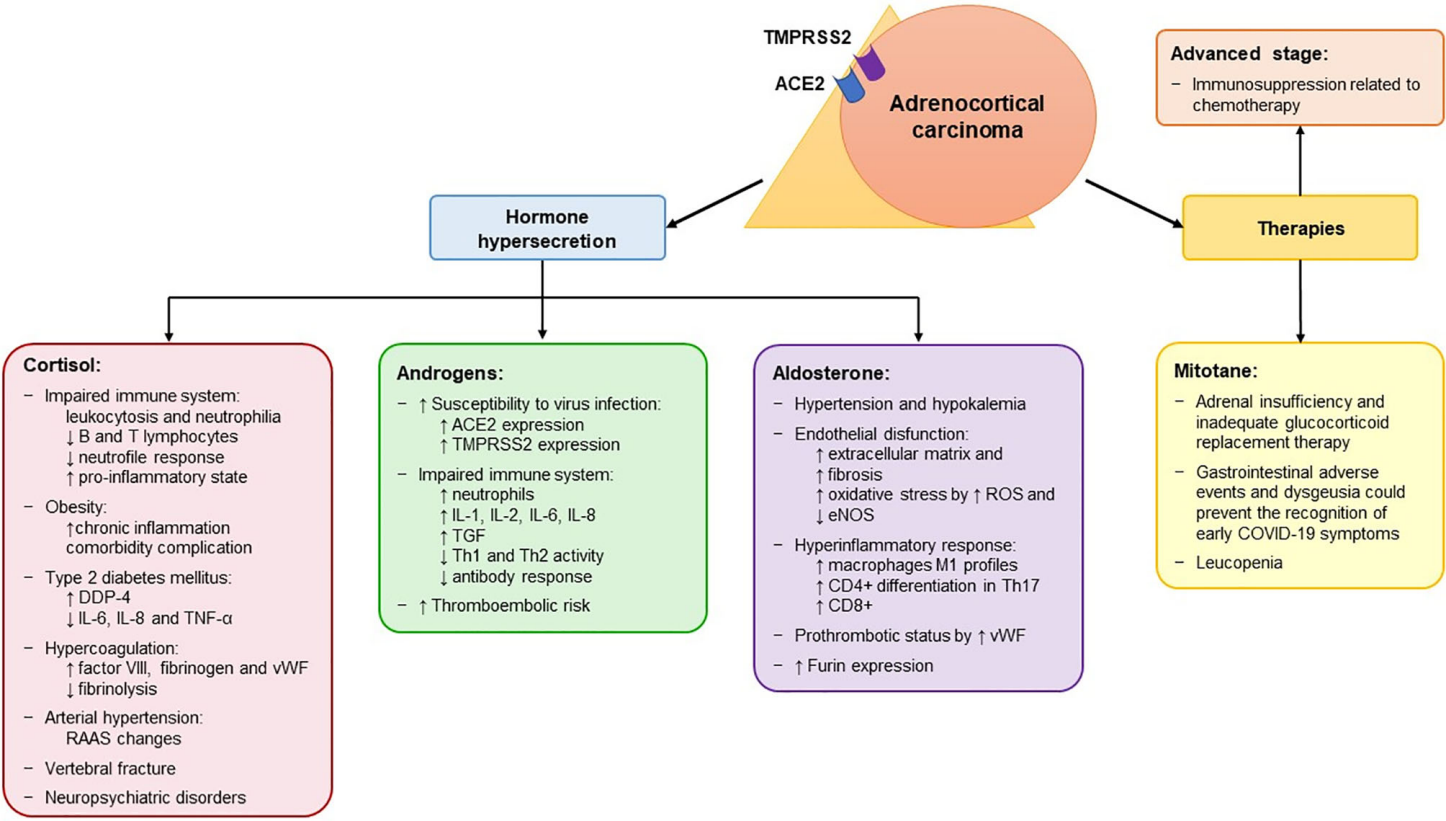
Potential mechanisms associated with an increased risk of contracting SARS-CoV-2 and with a more severe form of the disease in patients with adrenocortical carcinoma. Adrenocortical carcinoma (ACC) expresses both *ACE2* and *TMPRSS2* at mRNA levels, which encode the receptors that mediate the entry of SARS-CoV-2 into the host cell. The peculiarity of ACC is its hormone secretion (in 50-60% of patients), resulting in hypercortisolism, hyperandrogenism and hyperaldosteronism. In addition, it is treated with mitotane therapy, which is used both as adjuvant treatment and in advanced cases and induces adrenal insufficiency. The effects of the hormone secretion and of mitotane are listed in the corresponding box. In addition, ACC patients also present other cancer-related factors that are associated with a high risk of severe manifestations of SARS-CoV-2, including advanced stage and immunosuppression related to chemotherapy. ACE2, angiotensin converting enzyme 2; eNOS, endothelial nitric oxide synthase; ROS, reactive oxygen species; TMPRSS2, transmembrane protease serine 2; vWF, von Willebrand factor; ↑, increased; ↓, decreased; RAAS, Renin-Angiotensin-Aldosterone System; DDP-4, dipeptidyl peptidase 4; IL, interleukine; TGF, tumor growth factor; Th, T helper.

These cases highlight several observations (126). Given the features of ACC, including steroid hormone secretion and AI secondary to mitotane, ACC patients could harbor a higher risk of contracting SARS-CoV-2 and/or significant COVID-19 complications compared to other cancer patients (**Figure 3**). In addition, we observed *in silico*, for the first time, that the mRNA of *ACE2*, *TMPRSS2*, *CTSB*, *CTSL*, and *FURIN*, which encode the receptors and co-receptors that facilitate the entry of SARS-CoV-2 into the target cells, is expressed in ACC tissues (**Figure 2**). No difference in the expression of these genes was found between ACC, benign adrenocortical tumors and normal adrenal glands. These findings suggest that ACC might be a potential direct target of SARS-CoV-2, as has also been observed for normal adrenal glands.

The majority of ACCs are steroid-secreting tumors, mostly related to hypercortisolism (121). As mentioned above, CS is associated with immune system abnormalities, obesity, T2DM, hypercoagulation, arterial hypertension, vertebral fractures, and neuropsychiatric disorders, which could increase the risk of severe manifestations of SARS-CoV-2 (**Table 2** and **Figure 3**). Particularly, in ACC patients, T cell depletion is associated with glucocorticoid excess and T2DM is correlated with unfavorable prognosis (128, 129).

Cortisol secretion in ACC patients is often associated with hyperandrogenism. Evidence from the literature supports different plausible androgen-derived mechanisms driving clinical outcomes in COVID-19 (130, 131) (**Figure 3**). Firstly, androgens partly regulate the expression of the host cell receptor ACE2 and co-receptor TMPRSS2 (132, 133). Therefore, hyperandrogenism could lead to an upregulation of the expression of both ACE2 and TMPRSS2. Secondly, it has been shown that other co-receptors that enhance viral binding to ACE2, including furin, are downstream targets of the androgen receptors (132). Thus, hyperandrogenism may promote SARS-CoV-2 entry into cells by interacting with both ACE2 and co-receptors. These findings are also supported by the fact that androgen deprivation and anti-androgen treatment attenuated SARS-CoV-2 cellular entry *in vitro* and *in vivo* (133). Looking at the results of our *in silico* analysis (**Figure 2**), we observed that two of the four patients with reported hyperandrogenism had very high *ACE2* expression levels. The other outliers with very high *ACE2* levels were two patients with CS and two patients (including one man) with no hormone hypersecretion. No correlation was observed between hormone secretion and expression of the other investigated genes. However, since detailed information on hormone secretion is lacking for the investigated dataset (14), its results are difficult to interpret.

Androgens can also modulate immune response (130). There is evidence that androgens increase the levels of circulating neutrophils and of different interleukins, including IL-1, IL-2, IL-6, IL-8, and TGF-b, while decreasing Th1 and Th2 cell activity and antibody response to viral infections (131, 134). These effects could impair the immune response to SARS-CoV-2. Finally, treatment with testosterone enhances the platelet response and increases the number of thrombosis-related events in animal models (131). Thus, hyperandrogenism could exacerbate the already high thromboembolic risk observed in COVID-19 patients (135). All this evidence could explain the higher COVID-19 complication rate in patients with comorbidities associated with high androgen levels (132).

Conn’s syndrome is a rare manifestation in ACC. Although there is a lack of evidence on the role mineralocorticoids might play in the context of the COVID-19 pandemic, aldosterone might exacerbate the severity of SARS-CoV-2 in ACC patients (**Figure 3**). Aldosterone is the terminal hormone of the renin–angiotensin–aldosterone system (RAAS). By binding to the mineralocorticoid receptor (MR) in the epithelial cell of the distal nephron, it induces the reabsorption of sodium and excretion of potassium and hydrogen ions, causing water reabsorption and volume expansion. These effects explain why patients with Conn’s syndrome typically present hypertension and hypokalemia (136), which have also been observed in a large percentage of patients with COVID-19 (137). MRs are also found in non-epithelial tissues such as the myocardium, vascular smooth muscle cells, macrophages, fibroblasts, and adipocytes, where aldosterone mediates inflammation and affects energy metabolism (138). Hyperaldosteronism mediates endothelial dysfunction by increasing pulmonary, heart and kidney fibrosis (138, 139). It also increases reactive oxygen species levels and decreases the activity of endothelial nitric oxide synthase, leading to increased oxidative stress in epithelial cells (140). Aldosterone mediates several immune effects and promotes a hyperinflammatory response (141). Specifically, MR activation in immune cells causes a shift towards the M1 proinflammatory phenotype in macrophages, enhances the differentiation of CD4^+^ T lymphocytes into Th17 cells, and increases CD8^+^ cell cytotoxicity (138). In addition to these effects, aldosterone stimulates the release of vWF from endothelial cells, enhancing leukocyte adherence, platelet adhesion and the formation of microthrombi (142), which are frequently reported in COVID-19 patients (135). Finally, it promotes viral entry by increasing the activity of furin (143). All this evidence underlines the potentially harmful effects of mineralocorticoids in the setting of SARS-CoV-2 and points to the benefits of spironolactone and other MR-antagonists in COVID-19 patients. It is worth noting that spironolactone, unlike other RAAS inhibitors, also has a significant antiandrogenic effect (138, 143). The synergism of mineralocorticoid and androgen receptor blockade might therefore be of benefit for COVID-19 patients.

It is important to note that MRs can also be activated by high levels of cortisol. After binding to MRs, cortisol induces the exocytosis of Weibel-Palade bodies containing vWF and angiopoietin-2, which triggers ARDS by increasing capillary permeability (140). The beneficial effects of dexamethasone in COVID-19 patients could thus be explained not only by its anti-inflammatory action, but also by its inhibition of endogenous cortisol secretion. This prevents the cortisol-associated activation of MRs, since dexamethasone has almost no mineralocorticoid action. The combination of dexamethasone and spironolactone could therefore lead to the more efficient inhibition of MRs in COVID-19 patients (140).

Another important factor that might increase the risk of contracting SARS-CoV-2 in ACC patients is mitotane (**Figure 3**), which is usually used over long periods of time in ACC patients (144). In fact, all reported ACC cases were receiving mitotane when they tested positive for SARS-CoV-2 (126). Mitotane is the only approved drug in advanced ACC, and can be used as monotherapy or in combination with chemotherapy (121, 145–148). However, due to the high rate of recurrence after complete ACC resection, it is also used in adjuvant settings (149–151). Due to its direct cytotoxic effect on adrenocortical cells and its inhibition of steroidogenesis, mitotane induces several adverse events (121, 152). Generally, all patients treated with mitotane develop temporary AI, necessitating glucocorticoid replacement therapy. However, because glucocorticoid clearance is increased by mitotane, patients need higher doses of glucocorticoids than with other types of AI. As mentioned above, inadequate glucocorticoid replacement therapy could be associated with a higher risk of infection and an impaired early immune response to viral infections in AI patients. Mitotane often induces gastrointestinal effects such as diarrhea, nausea, vomiting, and lack of appetite, in addition to AI (149, 152). These symptoms could mask a SARS-CoV-2 induced gastrointestinal infection, which has been described in a subset of patients (153, 154), thus delaying the diagnosis of COVID-19. Another symptom that could lead to a delayed COVID-19 diagnosis is dysgeusia, which is frequently reported in COVID-19 patients (3) but also is an adverse event of mitotane (152). Leucopenia also occurs with mitotane (149, 152) and is associated with a severe course of COVID-19 and an increased mortality risk in cancer patients (122). Due to the potential misdiagnosis and increased risk of severe COVID-19 infection, we suggest the suspension of mitotane in cases of strongly suspected or confirmed SARS-CoV-2 infection until a negative nasopharyngeal swab is available or until clinical recovery. Moreover, steroid supplementation should be adjusted in cases of fever or other acute symptoms.

In addition to the particular features of ACC itself, ACC patients also present other cancer-related factors associated with a high risk of severe manifestations of SARS-CoV-2, including advanced stage disease and immunosuppression related to chemotherapy (**Figure 3**). The only ACC patient who died of COVID-19 had multiple pulmonary and hepatic metastatic lesions and presented with neutropenia and lymphopenia induced by temozolomide (126). It is important to underline that no significant difference was observed between ACC and other tumor types in terms of mortality rate due to COVID-19 (17% vs 27%, respectively) (126). Advanced stage, disease progression and antineoplastic therapy within 2-4 weeks prior to COVID-19 infection have been associated with a higher risk of severe and fatal outcome of COVID-19 in other cancer patients (122, 123, 125, 155). Because of the aggressiveness of ACC, which shows a 5-year overall survival of about 15% in metastatic disease (stage IV according to the European Network for the Study of Adrenal Tumors classification) (156), surgical and medical treatment should not be delayed. Therefore, in cases of suspected/confirmed ACC, a complete *en bloc* resection (if possible) should be attempted (157, 158).

Since lymphopenia secondary to chemotherapy is associated with an increase in COVID-19 mortality (122, 125), it seems reasonable to debate whether certain antineoplastic therapies should be delayed or discontinued. However, advanced ACC has an unfavorable prognosis and uncontrolled tumor progression is an independent risk factor for severe COVID-19. Moreover, there are discordant data in relation to the potential association of chemotherapy with a poor clinical outcome in cancer patients (159, 160). Taking this into consideration, we strongly recommend performing antineoplastic treatment in advanced ACC patients to achieve the best possible tumor response. Platinum-based chemotherapy in combination with etoposide and doxorubicin plus mitotane is the first line of treatment (121, 147). Given that platinum compounds are only modestly associated with myelosuppression, their use appears the most appropriate option, including during the COVID-19 pandemic (157).

## 6 Conclusion

The entire HPA axis could be a direct target of SARS-CoV-2, due to the expression of ACE2 receptors and TMPRSS2 co-receptors in the hypothalamus and pituitary and adrenal glands. Moreover, both impaired cortisol secretion and cortisol excess, as observed with AI and CS, as well as advanced or completely resected ACC under treatment with mitotane, may increase susceptibility to SARS-CoV-2 and the risk of severe forms of COVID-19. Patients with AI, CS or ACC are therefore a vulnerable patient population and their management should be interdisciplinary, if necessary through virtual meetings, in order to provide the best individualized patient management.

The evidence emphasizes the need to modify the management of these patients in the event of contracting SARS-CoV-2. In patients with AI, including cases secondary to surgery or medical treatment for CS or ACC, glucocorticoid replacement should be increased in relation to the severity of COVID-19 symptoms, in accordance with current European recommendations (39). Patients with active CS and SARS-CoV-2 might be best treated according to the ‘block and replace’ approach with steroidogenesis inhibitors (metyrapone or ketoconazole) and glucocorticoid replacement (hydrocortisone, dexamethasone or prednisolone) to prevent AI crises (77). Particular attention should be given to patients with ACC. Following the recommendations of endocrine surgeons (157, 158), tumor resection should not be postponed. Mitotane treatment and antineoplastic therapies should be suspended in the case of severe manifestations of SARS-CoV-2, but the start of the treatment should not be postponed in the absence of any symptoms of COVID-19 (125, 161).

Given the evidence discussed herein, we believe that patients with AI, CS or ACC, whether with active cancer or currently tumor-free, should be provided with priority access to COVID-19 vaccination to protect them from the high risk of severe infection. In patients with active CS, serum cortisol levels should be controlled prior to vaccination, as overt hypercortisolism might impair the immune response to the vaccine. Therefore, we recommend following the indications of the endocrinological societies, including the German Society of Endocrinology (https://www.endokrinologie.net/aktuelles-details/endokrinologische-krankheitsbilder-covid-19.php), which include these patients in the high priority groups.

## Author Contributions

BA designed and coordinated the study. IC, MD, and BA participated in the literature search and performed the selection of studies. IC wrote the paragraph on adrenal insufficiency. MD wrote the paragraph on Cushing’s syndrome. BA wrote the introduction and the paragraphs on adrenocortical carcinoma. UD, OK, FM, SH, and MF critically reviewed the manuscript and revised it for important intellectual content. All authors contributed to the article and approved the submitted version.

## Funding

This review was supported in part by the Deutsche Forschungsgemeinschaft (DFG) (project number 314061271 - TRR 205 and project number FA-466-5-1), the Clinician Scientist program RISE funded by the Else-Kröner-Fresenius-Stiftung & the Eva Luise und Horst Köhler Stiftung, and the Deutsche Krebshilfe (project number #70113526).

## Conflict of Interest

The authors declare that the research was conducted in the absence of any commercial or financial relationships that could be construed as a potential conflict of interest.

## Publisher’s Note

All claims expressed in this article are solely those of the authors and do not necessarily represent those of their affiliated organizations, or those of the publisher, the editors and the reviewers. Any product that may be evaluated in this article, or claim that may be made by its manufacturer, is not guaranteed or endorsed by the publisher.
